# Quantum Valley Hall Effect‐Based Coupler with Continuously Tunable Transmission for Topological Information Communication

**DOI:** 10.1002/advs.202506732

**Published:** 2025-06-29

**Authors:** Keita Funayama, Kenichi Yatsugi, Hiroya Tanaka, Hideo Iizuka

**Affiliations:** ^1^ Toyota Central R&D Labs., Inc. Nagakute Aichi 480–1192 Japan

**Keywords:** digital data transfer, elastic waveguide, micro electromechanical systems, topological insulator, tunable coupler

## Abstract

Topological insulators have taken classical wave systems to the next level by their unique characteristics such as highly efficient, robust, and unidirectional wave propagation. As a step further, tunability in fundamental building blocks such as couplers is desired toward topological integrated circuits and communication systems. However, most of existing topologically protected couplers exhibit a constant transmission coefficient within the bandgap, and achieving continuously tunable transmission coefficients remains challenging. Here it is experimentally showed that a four‐port coupler consisting of quantum valley Hall effect‐based waveguides can exhibit continuously tunable transmission coefficients from one input port to each of the two output ports over the bandgap, while the rest port is consistently isolated by the topological protection. Further topologically protected digital signal transfer is demonstrated with frequency shift keying modulation in the coupler, revealing the data‐transfer capability close to the theoretical limit established in the digital communication scheme. These results pave the way for topological data transfer and signal processing.

## Graphical abstract

The quantum‐valley‐Hall‐based bridge boundary has the frequency dependent wave localization. Utilizing the frequency dependency, this article experimentally shows the micro‐electromechanical‐systems‐based topological coupler with continuously tunable transmission coefficients by the frequency of interface modes within a single bulk bandgap. Furthermore, it is demonstrated that the topological wave coupler works as a signal receiver, filter, and demodulator for digital communication.

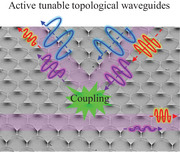

## 1 Introduction

The discovery of topological phases of matters has offered unique wave phenomena such as robust and one‐way propagation along topologically protected edge structures and interfaces.^[^ ^1^, ^2^, ^3^ ^]^ Such topologically protected wave control has been applied to classical wave systems in several fields such as optics,^[^ ^4^, ^5^, ^6^, ^7^, ^8^, ^9^, ^10^, ^11^, ^12^, ^13^ ^]^ electronics,^[^ ^14^, ^15^, ^16^, ^17^, ^18^ ^]^ acoustics,^[^ ^19^, ^20^, ^21^, ^22^, ^23^, ^24^, ^25^ ^]^ and elastodynamics.^[^ ^26^, ^27^, ^28^, ^29^, ^30^, ^31^, ^32^, ^33^, ^34^, ^35^, ^36^, ^37^, ^38^, ^39^ ^]^ In addition, a topological scheme having high affinity with established conventional technologies is beneficial for practical applications based on wave propagation. Consequently, topological nature is a promising candidate for highly efficient and robust transfer of signals and energies.

Due to the unique characteristics, integration of topological waveguides has the potential for lossless and robust circuit systems, namely, topological circuit systems driven by several types of propagating waves. Similar to modern circuit systems, integration of circuit elements is desired in topological circuit systems for exhibiting multifunctionality. Topological circuit elements have been widely explored, including topological couplers,^[^ ^40^, ^41^, ^42^, ^43^ ^]^ wave separators,^[^ ^44^, ^45^, ^46^, ^47^ ^]^ and interface connections with classical waveguides.^[^ ^48^, ^49^ ^]^ Especially, coupler is a key fundamental building block for integrated circuits^[^ ^50^, ^51^ ^]^ because any linear optical functionalities can be realized by cascading couplers.^[^ ^52^ ^]^ In topological wave couplers, several schemes for engineering transmission coefficients have been presented by using topological structures with dual bandgaps^[^ ^53^, ^54^ ^]^ and combination of different topological characteristics.^[^ ^42^, ^45^, ^55^, ^56^, ^57^ ^]^ However, in those approaches, the transmission coefficients are almost constant for each bandgap. It is a challenge for the control of transmission coefficients within a single bandgap, due to the intrinsic topological nature of the robustness. Thus, topological couplers with continuous and active tunability within a single bandgap have not been reported yet. As indicated in modern integrated circuits for high‐performance electronic devices and multifunctional communication applications, circuit elements with continuously tunable output characteristics can exhibit substantially advanced functionality of such systems. Thus, combining topological protection and continuously tunable transfer characteristics has the potential to become a game changing strategy for developing topological waveguides‐based integrated circuits.

Our previous work has found that a bridge boundary based on the quantum valley Hall effect (QVHE) can possess frequency dependent wave profiles.^[^ ^58^ ^]^ Inspired from the finding, in this work, we experimentally demonstrate a topologically protected microelectromechanical systems (MEMS)‐based wave coupler having continuously tunable transmission coefficients in the frequency spectra within a bandgap. The topological coupler consists of two adjacent topological waveguides, and the coupling between them can be engineered by the frequency‐dependent wave profiles. Such functional topological couplers will contribute to bridging the gap between fundamental physics and practical engineering.

Micro/Nano‐electromechanical systems (MEMS/NEMS) function as excellent signal processing devices such as band filters, converters, and receivers for radio waves because the sizes of MEMS/NEMS correspond to radio wavelengths.^[^ ^59^, ^60^, ^61^, ^62^, ^63^, ^64^, ^65^, ^66^ ^]^ Those mechanical systems have been attracting attention for downsizing communication devices, implementing signal processing function from reception to demodulation in one device, while suppressing signal dissipation and improving the reception efficiency in those systems have remained challenges yet. Using the topological nature of robust and one‐way wave‐propagation, topological MEMS couplers are highly compatible with digital communication, which is one of the significant technologies for the current human society. As an illustration, we present a demonstration of topologically protected data transfer through the frequency‐dependent topological coupler with a frequency shift keying (FSK) scheme. In our demonstration, the MEMS‐based coupler includes functionalities of reception, filtering, and demodulation of electric FSK signals. As the information communication system, we experimentally evaluate the maximum mutual information, i.e., channel capacity, in the topological coupler. Combining with signal processing for improving detection rate, we verify that the coupler has a data‐transfer capability close to the theoretical limit established in the digital communication scheme.

## 2 Results

### 2.1 Topological Coupler with Continuously Tunable Transmission Coefficients

So far, our previous work has clarified that there are frequency‐dependent and ‐independent boundaries for QVHE‐based topological boundaries.^[^ ^58^ ^]^ Herein, we investigate the topological couplers consisting of the frequency‐dependent and ‐independent boundaries, respectively.

**Figure 1:**
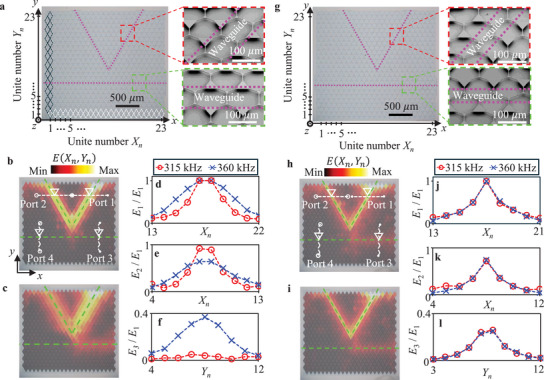
Frequency‐dependent and ‐independent topologically protected couplers, and their field profiles. a) Top‐view of the microelectromechanical systems (MEMS)‐based frequency‐dependent topological coupler consisting of 23 × 23‐unit cells. The magenta lines represent the topological waveguides. Highlighted scanning electron microscope (SEM) images by red and green lines indicate the enlarged views of the V‐shaped and straight waveguides, respectively. b,c) The square of the z component of displacement *E*(*X_n_*, *Y_n_*) profiles on the 2D MEMS structure in (a) for low (315 kHz) (b) and high (360 kHz) (c) frequencies. d–f) 1D field profiles at ports 1 (d), 2 (e), and 3 (f) in (a). g) Top‐view and highlighted SEM imgages of the MEMS‐based frequency‐independent topological coupler. h,i) The square of the z component of displacement *E*(*X_n_*, *Y_n_*) profiles on the 2D MEMS structure in (g) for low (315 kHz) (h) and high (360 kHz) (i) frequencies. j–l) 1D field profiles at ports 1 (j), 2 (k), and 3 (l) in (g).

We first consider a frequency‐dependent topologically protected elastic wave coupler, as shown in **Figure** **1**a. The coupler consists of 23 × 23‐unit cells with triangular two‐sublattices formed by a 2D Si beam. The spatial symmetry in the unit cell is different by inverting the size of the two‐sublattices on the both sides of the boundaries highlighted by the magenta lines. The enlarged scanning electron microscope (SEM) images indicate that the boundaries are composed of the neighboring two small triangular sublattices. This symmetry breaking allows topologically protected wave propagation based on the QVHE scheme. Due to the C_3_ symmetry of the unit cell, the direction of the topological boundary is restricted to a 120° interval. Thus, we fabricate the straight and V‐shaped topological elastic waveguides to achieve the coupler structure.

Electrostatically exciting transverse waves from the right upper edge of the V‐shaped waveguide, we measure the square of the z component of the displacement at ports 1 (*E*_1_), 2 (*E*_2_), 3 (*E*_3_), and 4 (*E*_4_) (Figure 1b) to investigate transmission properties of the coupler (see Experimental Section for the details). Figure 1b,c shows the 2D profiles of the square of the z component of displacement, *E*(*X_n_*, *Y_n_*), on the entire structure at low (315 kHz) and high (360 kHz) frequencies for interface modes, respectively. For the low‐frequency excitation (Figure 1b), the elastic wave dominantly propagates along the V‐shaped waveguide from port 1 to port 2, and the V‐shaped waveguide does not couple with the straight waveguide because of the strong localization of the elastic wave in the V‐shaped waveguide. On the other hand, for the high frequency excitation (Figure 1c), we see that the elastic wave profile broadens, resulting in wave propagation from port 1 to port 3 along the straight waveguide as well as the V‐shaped waveguide due to the coupling between the two topological waveguides. The frequency‐dependent wave localization above induces transition from decoupling to coupling between the two waveguides.

We clarify the frequency‐dependent wave localization by visualizing the field profile at each port. Indeed, the input field profiles at port 1 show sharp (red circles) and broad (blue crosses) energy distributions for low and high frequencies, respectively (Figure 1d). For the low frequency, the normalized energy profile at port 2 gives more than 90% kinetic energy propagation from port 1 to port 2 along the V‐shaped waveguide (Figure 1e) while negligibly small propagation from port 1 to port 3 by topological protection (Figure 1f). For the high frequency, we emphasize that the field profiles at ports 2 and 3 ensure wave propagation to both ports 2 and 3 by coupling between the two waveguides (Figure 1e,f). Note that the propagating wave along the V‐shaped waveguide is spin‐momentum locked for the interface modes. The propagating direction of the unidirectional wave depends on the pseudo‐spin. The pseudo‐spin of the unidirectional wave from port 1 to port 2 corresponds to that from port 4 to port 3. Thus, in our experimental setup, port 4 becomes an isolation port when the V‐shaped waveguide couples with the straight waveguide for the interface mode (Figure S1, Supporting Information).

As shown in Figure 1g–l, we consider a frequency‐independent topologically protected elastic wave coupler having a different boundary geometry. For the topological boundary, we see that topologically protected interface modes have almost constant localization characteristics within a bandgap. When the right‐upper edge of the V‐shaped waveguide is excited at low (315 kHz) and high (360 kHz) frequencies, respectively, the coupler composed of the V‐shaped and straight topological waveguides exhibits the similar 2D filed profiles at both frequencies (Figure 1h,i). The energy distributions at ports 1 (Figure 1j), 2 (Figure 1k), and 3 (Figure 1l) clearly indicate constant localization in the waveguides regardless of the frequency, resulting in constant transmission coefficients over the entire frequency spectra.

**Figure 2:**
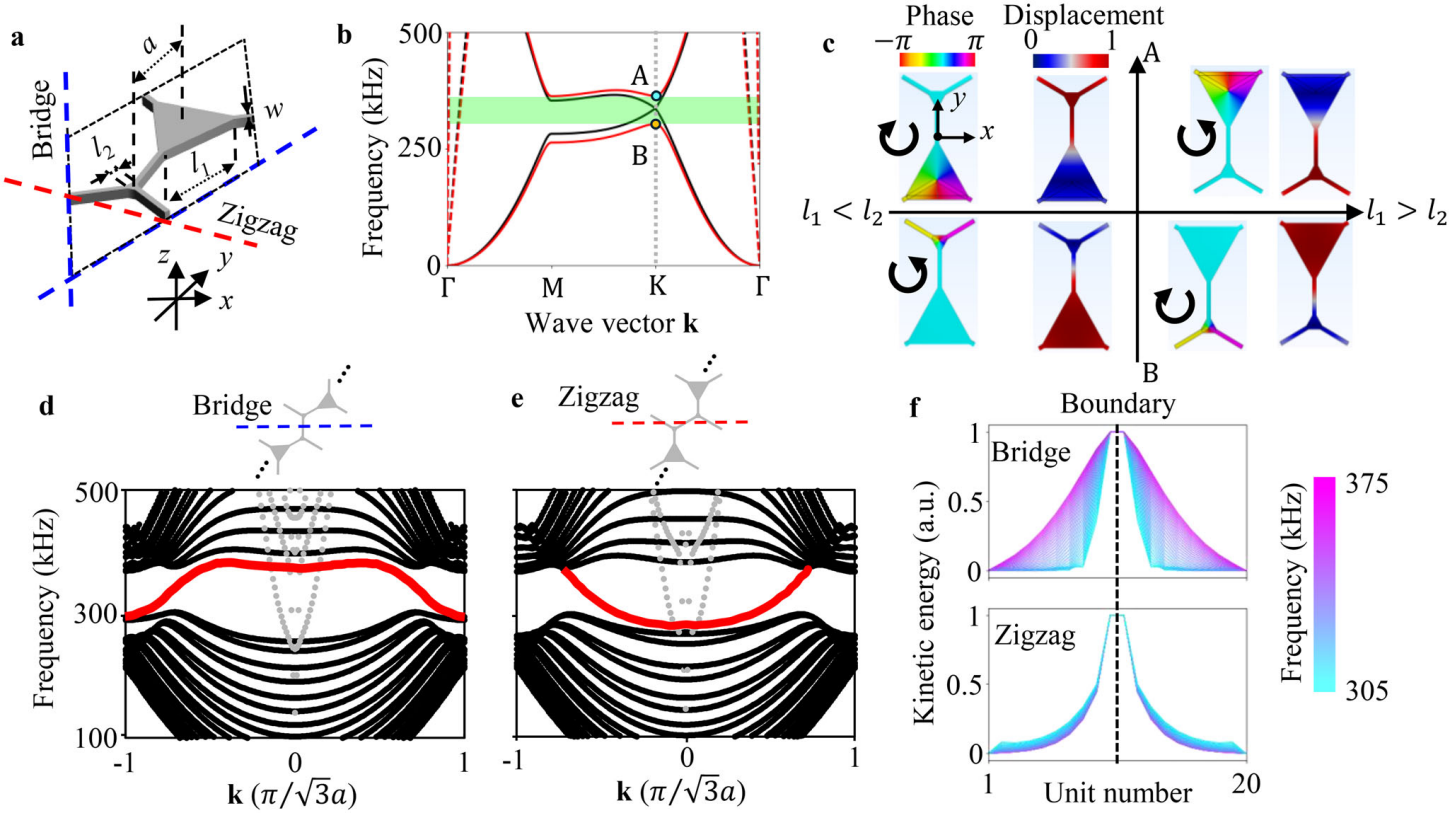
Quantum valley Hall effect‐based topological phononic unit cell and supercells. a) Schematic of the unit cell composed of two inequivalent triangular sublattices. Here, *a* is the distance between the centers of the two sublattices, *l*_1(2)_ is the side length of the triangular plates, and *w* is the width of the beam. The blue and red lines represent the boundaries of bridge and zigzag, respectively. b) Dispersion diagrams of infinite periodic unit cells for (*l*_1_, *l*_2_) = (50 µm,  15 µm) (red lines) and (*l*_1_,*l*_2_) = (32.5 µm,  32.5 µm) (black lines). The solid and broken lines represent transverse and longitudinal waves, respectively. The green shaded region represents the bandgap. Two modes A (cyan plot) and B (orange plot) appear at K point by breaking degeneracy. c) Displacement and phase distributions of modes A and B for (*l*_1_, *l*_2_) = (50 µm,  15 µm) (*l*_1_ > *l*_2_) and (*l*_1_, *l*_2_) = (15 µm,  50 µm) (*l*_1_ < *l*_2_). d,e) Dispersion diagrams of the supercell having the bridge (d) and zigzag (e) boundaries. The red lines in the dispersion diagrams indicate topological interface modes. f) 1D field profiles of the supercells having the bridge and zigzag boundaries for interface modes within the bandgap of (d) and (e) as frequency is varied from 305 to 375 kHz.

The frequency‐dependent and ‐independent couplers have been obtained by designing a two‐sublattice‐based unit cell and selecting the bridge (blue lines) and zigzag boundaries (red line) as shown in **Figure** **2**a, respectively. The unit cell with the asymmetric sublattices of the large‐ (*l*_1_ = 50 µm) and small‐triangular (*l*_2_ = 15 µm) plates has a bandgap (green shaded region) in the dispersion diagram as shown in Figure 2b in terms of transverse waves. As a reference, the solid black line represents the dispersion diagram of transverse waves for the symmetric two‐triangular sublattices (*l*_1_ = *l*_2_ = 32.5 µm). Note that the broken lines indicate the dispersion diagram of longitudinal waves, and such longitudinal waves can be sufficiently suppressed by exciting the structure along the *z*‐axis in Figure 2a. For the asymmetric two sublattices, there are two modes above (A) and below (B) the bandgap at K point in Figure 2b. Figure 2c shows the displacement and phase profiles in the unit cell at points A and B. For *l*_1_ > *l*_2_, mode A [B] shows the counter‐clockwise [clockwise] phase and maximum displacement at (*x*,  *y*) = (0,   − *a*/2) [(*x*,  *y*) = (0,  *a*/2)]. For *l*_1_ < *l*_2_, mode A [B] exhibits the counter‐clockwise [clockwise] phase and maximum displacement at (*x*,  *y*) = (0,  *a*/2) [(*x*,  *y*) = (0,   − *a*/2)]. This band inversion indicates that the boundary between the unit cells for *l*_1_ > *l*_2_ and *l*_1_ < *l*_2_ allows an existence of QVHE‐based topological interface modes.

We prepare supercells with the bridge and zigzag boundaries to investigate the topological interface modes as shown in Figure 2d,e. For both boundaries, each of the upper (*l*_1_ > *l*_2_) and lower (*l*_1_ < *l*_2_) regions in the supercells is composed of 10‐unit cells, respectively. The dispersion diagrams for both supercells similarly have topological interface modes (red lines) crossing the bandgap from 305 to 375 kHz between the bulk bands (black plots) in terms of transverse waves. We select the experimental excitation frequencies (315 and 360 kHz) in Figure 1 by referring with the frequency range between the numerical bandgap in Figure 2d,e. Note that modes of longitudinal waves (gray plots) crossing the bandgap can be ignored by exciting the supercells along the *z*‐axis in Figure 2a, as mentioned above.

Based on the experimental observation in Figure 1, frequency‐dependent decay of the kinetic energy away from the bridge and zigzag boundaries is investigated in the range of the bandgap. Figure 2f shows the comparison of field profiles for the bridge and zigzag boundaries as frequency is varied from 305 to 375 kHz. We clearly see that the profiles *E*_1_/*E*_1_, *E*_2_/*E*_1_, and, *E*_3_/*E*_1_ for the bridge boundary broaden with increasing the frequency in the bandgap. On the other hand, the wave energy profiles *E*_1_/*E*_1_, *E*_2_/*E*_1_, and, *E*_3_/*E*_1_ for the zigzag boundary exhibit constant localization over the bandgap, resulting in frequency independence.

**Figure 3:**
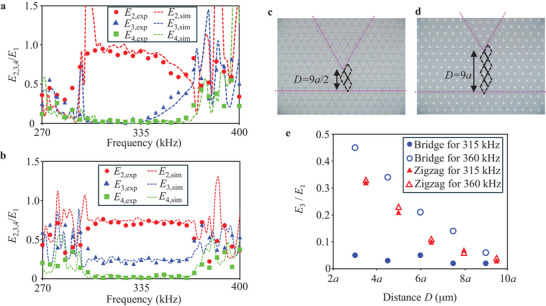
Frequency‐ and distance‐dependencies of the transmission coefficients in topological couplers. a,b) Frequency spectra of the kinetic energy at ports 2, 3, and 4 for the couplers consisting of bridge (a) and zigzag (b) boundaries. c,d) Photomicrographs of the topological couplers having 3‐unit cells (c) and 6‐unit cells (d) between the V‐shaped and straight waveguides (broken magenta lines). e) Transmission coefficients at port 3 in the frequency‐dependent (circle plots) and frequency‐independent (triangular plots) couplers as a function of distance *D*.

Near the corner of the V‐shaped waveguide, the overlap of localized wave profiles between the V‐shaped and straight waveguides generates energy exchange between them, thereby, the variation of the overlap arising from frequency‐dependent localization induces the tunability in the coupler. To validate continuously tunable transmission coefficients in the coupler, we experimentally investigate the frequency spectra of the kinetic energy at ports 2, 3, and 4 normalized by the kinetic energy at port 1 around the bandgap (**Figure** **3**a). Circular, triangular, and square symbols in Figure 3a indicate experimentally measured *E*_2_/*E*_1_, *E*_3_/*E*_1_, and *E*_4_/*E*_1_, respectively. Within the bandgap from ≈300 to 370 kHz, the sum of the output kinetic energy at ports 2 and 3 accounts for more than 90% of the input energy at port 1, ensuring highly efficient energy transport along the topologically protected V‐shaped and straight waveguides. As expected from the frequency‐dependent localization in Figure 2f, we find that the transmission coefficients at ports 2 and 3 continuously decrease and increase with increasing the frequency, respectively. The consistent energy isolation at port 4 is an additional evidence of topological protection in the coupler. The experimental results (symbols) are in good agreement with the numerical results of *E*_2_/*E*_1_ (red line), *E*_3_/*E*_1_ (blue line), and *E*_4_/*E*_1_ (green line). In our coupler, the transmission coefficients can be continuously tuned from 0.95 to 0.59 for port 2 and from 0.02 to 0.40 for port 3, respectively.

Similarly, we investigate transmission characteristics of the frequency‐independent coupler composed of the zigzag boundaries shown in Figure 1g. Figure 3b shows the frequency spectra of *E*_2_/*E*_1_ (red circles), *E*_3_/*E*_1_ (blue triangles), and *E*_4_/*E*_1_ (green squares) for the coupler around the bandgap. Due to topological protection, the total kinetic energy of *E*_2_ and *E*_3_ exceeds 90% of input energy *E*_1_ in the bandgap from 300 to 370 kHz. On the other hand, the V‐shaped and straight waveguides keep constant transmission coefficients 0.71 (port 2) and 0.23 (port 3) for the interface mode in the entire bandgap, with port 4 being isolated. Comparing the results in Figure 3a,b, we clearly see that the frequency responses of the two couplers can be largely different by engineering the boundary geometries, i.e., frequency‐dependent and ‐independent spectra over the bandgap.

As a conventional approach, transmission coefficients in topological couplers can only be discretely designed by the spatial distance between two adjacent waveguides.^[^ ^43^, ^67^ ^]^ We investigate the relationship between continuous and discrete control of transmission coefficients. Figure 3c,d shows two examples of couplers having 3‐ and 6‐unit cells between the V‐shaped and straight waveguides, respectively. The distance between the two topological waveguides is expressed as *D* = (3*n*/2)*a* and *D* = (3*n* + 1/2)*a* for the bridge and zigzag boundary‐based couplers, respectively, where *a* is the distance between the centers of the two sublattices in the unit cell along the *y*‐direction (Figure 2a), and *n* is the number of the unit cells between the two waveguides as highlighted by the black rhombi in Figure 3c,d.

Figure 3e shows transmission coefficient *E*_3_/*E*_1_ at port 3 as a function of *D*. We see that *E*_3_/*E*_1_ is enhanced with decreasing *D* for the bridge boundary‐based coupler excited by high frequency (blue open‐circles). On the other hand, for low frequency, the sharp wave confinement discussed above suppresses the coupling by the discrete design, resulting in almost constant values of *E*_3_/*E*_1_ in the range of *D* (blue filled circles). Thus, the combination of the discrete design and continuous tunable localization characteristics allows to expand the range of continuous tunability of transmission coefficients in our couplers, e.g., *E*_3_/*E*_1_ is largely varied from 0.05 to 0.45 at *D*  =  3*a*. For the zigzag boundary‐based couplers, the transmission coefficients increase with decreasing *D* similar to the bridge boundary‐based couplers. On the other hand, the transmission coefficients keep constant values for the same *D*, independent of the frequency (red filled and open‐triangles). Contrasting with the bridge boundary‐based couplers, transmission coefficients in the zigzag boundary‐based couplers are only controlled discretely by the design of *D* within the bandgap. Note that we use the structure having the largest on/off ratio, i.e., 11.3 at *D*  =  9*a*/2, for a demonstration of signal transfer shown later.

### 2.2 Topologically Protected Digital Signal Transmission

We have so far provided the topologically protected coupler having continuous tunability of the transmission coefficients in the frequency spectra, comparing with the conventional topological coupler without frequency dependence. Tunable couplers are one of the key fundamental building blocks for high‐performance circuits and multifunctional systems since all linear functionalities in integrated circuits can be realized by combinations of couplers.^[^ ^52^ ^]^ Thus, the tunable coupler we provide is crucial to achieve practical topological systems such as topologically protected data transfer and signal processing. As an example of digital signal transfer, the continuously tunable coupler enables filtering of signals multiplexed by finely discretized frequency within the bandgap. Due to the frequency dependence of our topological coupler, frequency modulation is more suitable than other modulations. Here, we demonstrate signal processing from reception to demodulation of digital signals modulated by binary‐FSK with the topological coupler.

**Figure 4:**
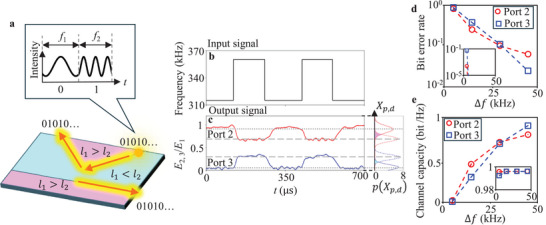
Digital data transfer in the topological coupler and evaluation of transfer capability. a) Schematic of transmission of digital signals modulated by frequency shift keying with the frequency‐dependent topological coupler. b) Frequency transition of the input signal at port 1 as a function of time. c) Output displacement at ports 2 and 3 as a function of time. Based on the averages and variances of the demodulated signal, we also calculate the probability density functions *p*(*X*_*p*, *d*_) for low (solid lines) and high (broken lines) frequencies. d) Bit error rate (BER) evaluated by experimental output signals at ports 2 and 3 in the frequency‐dependent topological coupler. e) Channel capacity of the frequency‐dependent topological coupler. The insets of (d) and (e) represent the BER and channel capacity calculated by eliminating the effect of frequency transitions, respectively.

**Figure** **4**a shows the schematic of our experimental setup for topologically‐protected digital signal transfer. We use FSK for signal modulation. Two discrete frequencies *f*_1_ and *f*_2_ (*f*_1_ < *f*_2_) correspond to digital signals 0 and 1, respectively. As observed in the frequency‐dependent transmission coefficients of the coupler in Figure 3a, we expect that the input digital signals at port 1 are demodulated to kinetic energy and then transmitted to ports 2 and 3 with a tunable ratio. In other words, our coupler is an all‐in‐one device playing the roles of signal transmission, signal separator, and frequency filter for FSK‐based communication. Figure 4b shows the frequency of the input signal as a function of time. We set *f*_1_ = 315 kHz and *f*_2_ = 360 kHz for the experimental demonstration. Figure 4c shows the output signals of *E*_2_/*E*_1_ (red line) and *E*_3_/*E*_1_ (blue line) at ports 2 and 3. Output signal *E*_2_/*E*_1_ (*E*_3_/*E*_1_) transits between 0.93 and 0.61 (0.05 and 0.35) when the frequencies of the input signals shift between 315 and 360 kHz. We see that the transition of the output signals occurs, immediately following the shift of the input signals. The sum of *E*_2_/*E*_1_ and *E*_3_/*E*_1_ keeps over 0.92 in the range of time, ensuring highly efficient digital signal transfer through the V‐shaped and straight topological waveguides. Note that the zigzag‐based coupler indicates constant output signals at ports 2 and 3 regardless of the frequency of the input signal (Figure S2, Supporting Information). Utilizing the average $\mu_{p,d}=\left(1/N\right)\sum_{n=0}^{N}\left\{E_{p,d}\left(n\right)/E_{1}\left(n\right)\right\}$ (*N*: number of data points) and variance $\sigma_{p,d}^{2}$ for each of the output signals, we calculate the probability density, which is expressed by $p\left(X_{p,d}\right)=\left(1/\sqrt{2\pi }\sigma_{p,d}\right)\exp\left\{-{\left(X_{p,d}-\mu_{p,d}\right)}^{2}/2\sigma_{p,d}^{2}\right\}$, as shown in the right‐hand side of Figure 4c, where *p*(= 2,  3) is the port number, *d*(= 0,  1) is a binary value of digital signal, and *X*_*p*,*d*_ (≥ 0) is the variable parameter along the axis of *E_p_*/*E*_1_. The solid and broken lines represent *p*(μ_*p*, 0_) and *p*(μ_*p*, 1_), respectively. The shaded regions by magenta and cyan indicate profile overlaps. Each of the overlap regions for *p*(*X*_2,*d*_) and *p*(*X*_3,*d*_) accounts for 12.8% and 5.04% of the total probability, respectively. Such overlaps cause communication errors, degrading the performance of data transfer.

Performance of digital communication systems has been usually evaluated in terms of bit error rate (BER). In Figure 4d, we show the BER obtained from the experimental results as a function of Δ*f* = *f*_2_ − *f*_1_ (see Method for the details), where *f*_1_ is varied at 350, 345, 330, and 315 kHz, with *f*_2_ = 360 kHz fixed. We find that the erroneous detection rate decreases with increasing Δ*f* at both ports 2 (red circles) and 3 (blue squares). Propagating elastic waves are demodulated as vibration amplitudes *A*_1_ and *A*_2_, corresponding to the signal frequencies of *f*_1_ and *f*_2_ by the frequency dependent coupler. Thus, Δ*f* is converted to Δ*A* = *A*_2_ − *A*_1_ through the MEMS‐based topological coupler. The vibration amplitude Δ*A* affects the signal to noise ratio, resulting in change of BER by Δ*f*.

Further, we investigate the data transfer capability of the topological coupler by calculating the maximum mutual information named as channel capacity. In the field of information theory, channel capacity has been introduced to measure the rate at which digital information can be reliably transmitted over a channel.^[^ ^64^, ^68^, ^69^ ^]^ Figure 4e shows the channel capacity calculated from the measured probability density at ports 2 (red circles) and 3 (blue squares) (see Experimental Section for the details). In binary bit data transfer with FSK, the theoretical limit of channel capacity is calculated to be 1 bit/Hz, which is the maximum speed and efficient transfer with the bandwidth.^[^ ^64^, ^68^, ^69^ ^]^ We see that the capacities at ports 2 and 3 increase up to 0.9 and 0.8 bit/Hz, respectively, with increasing Δ*f*. Note that the BER and channel capacity are calculated after demodulating from Δ*f* to the difference in the kinetic energy, thereby, the BER and channel capacity, which have been independent of Δ*f* in conventional FSK scheme, depend on Δ*f*. Contrasting with conventional couplers, where large reflection would occur in such sharp‐bended waveguide, our topological coupler has suppressed back scattering and reflection, resulting in the high channel capacity.

## 3 Discussion

As provided above, our proposed frequency‐dependent coupler has allowed topologically protected digital data transfer. Herein, we consider a signal processing scheme to further approach the theoretical limit of the channel capacity. For the mechanical vibration‐based signal processing, the vibration transition time is affected by a non‐negligible mass.^[^ ^64^, ^70^, ^71^ ^]^ In the MEMS‐based coupler, we find that output signals in Figure 4c have a slope of d(*E*_2, 3_/*E*_1_)/d*t* at the timing for switching the input signal frequency from *f*_1_ to *f*_2_ and from *f*_2_ to *f*_1_. The slope may arise from the transition of the mechanical vibration of the 2D structure, and induce the overlaps of *p*(*E*_*p*,*d*_). Accordingly, in practical systems, we expect to decrease the BER by signal processing such as the elimination of signals during the transition. Indeed, the calculated BER by eliminating the frequency transition region is drastically improved as shown in the inset of Figure 4d. The BER for Δ*f* > 5 kHz is ensured to be lower than the order of 10^−5^, considering the number of experimental data points, i.e., *N*  =  3.25  × 10^7^. As the results of low BER for Δ*f* > 5 kHz, the inset in Figure 4e indicates that the maximum mutual information, i.e., channel capacity, approaches the theoretical limit of the data‐transfer capability. This proves that the topological coupler received and demodulated the incoming FSK signal at an ideal efficiency in the range of Δ*f*. The high channel capacity indicates that the slope during signal transition is the dominant error factor, with negligible effect from external and inherent noise. Thus, combining with an appropriate signal processing scheme, our topological coupler allows highly efficient and accurate data transfer close to the upper bound of information theory. According to the bandwidth and channel capacity, the transmission speed limit of our coupler‐based communication system is 65 kbps for binary digital modulation.^[^ ^72^ ^]^ It would be interesting to explore high speed transmission through high‐frequency operation by downsizing the unit cell and multi‐valued digital modulation. Note that the bandwidth in our topological coupler corresponds to the width of the bulk bandgap, i.e., 65 kHz.

In summary, we have experimentally demonstrated a topologically protected coupler with continuous tunability of transmission coefficients. The transmission coefficient tunability within the bandgap has been achieved by the frequency‐dependent wave localization on the QVHE‐based bridge boundaries. The transmission tunability range has been extended by the combination with the discrete coupling design based on the distance between the two waveguides. As a demonstration of topological data transfer and signal processing using the topological coupler, we have presented a highly efficient and secure digital‐signal transfer modulated by FSK, and evaluated the channel capacity that is close to the theoretical limit. Our results in this work open up topologically protected integrated and functional circuits toward topological data transfer and signal processing.

## 4 Experimental Section

### Numerical Simulations

Numerical models were prepared in this study by using the structure mechanical module in COMSOL Multiphysics 6.2. All the models consist of single‐crystal silicon structures with default material parameters in COMSOL, i.e., elastic modulus was 170 GPa, mass density was 2329 kg m^−3^, and Poisson's ratio was 0.28. At the cross sections of the connecting beams of the unit cell, the periodic boundary condition was set with Floquet periodicity to calculate the dispersion diagram in Figure 2b. To calculate the dispersion diagrams in Figure 2d,e, supercells were build consisting of 20‐unit cells with the bridge and zigzag boundaries at the center of the supercells, respectively. The 20‐unit cells arranged in a row were given infinite periodicity by setting the periodic boundary condition at the cross section of the fine beams connecting to the neighboring rows. For calculating the numerical results in Figure 3a,b, the numerical structures having the V‐shaped and straight waveguides were also build in Figure 1. To obtain unidirectional wave propagation along the waveguides, the surfaces on the unit cells of (*X_n_*, *Y_n_*) = (19,  22),  (19,  23), and (20,  22) were excited with a phase difference of 2π/3 in the counter‐clockwise direction. The numerical kinetic energy was calculated by the volume integration in each unit cell.

### Sample Fabrication and Experimental Details

The fabrication steps for MEMS‐based topological waveguides were as follows, which were reported in the previous works.^[^ ^43^, ^58^ ^]^ As a first step, a silicon on insulator substrate was cleaned in a piranha solution (4:1 mixture of H_2_SO_4_ and H_2_O_2_) and deionized (DI) water. The thicknesses of the silicon device layer, silicon dioxide layer, and silicon substrate layer are 1, 10, and 470 µm, respectively. Secondary, the OAP and electron beam resist (ZEP520A, Zeon Corporation) were coated by spin coating of 5000 and 600 rotation per minute (rpm), respectively. After post‐baking at 170 °C for 5 min, the pattern of the 2D structure was drawn by electron beam (EB) lithography (JEOL, JBX‐6300FS). Then, the drawn patterns were developed in ortho‐xylene for 2 min at 23 °C At the EB lithography, patterns of alignment marks were simultaneously drawn for the driving electrode described later. The device layer was etched by reactive ion etching with Bosch process (MUC‐21 ASE‐Pegasus, Sumitomo Precision Products) except for the area covered by the resist. After washing the substrate in the piranha solution and DI water, the OAP and photo resist (THMR‐iP3100MM, Tokyo Ohka Kogyo Co., Ltd.) were spin‐coated on the silicon substrate layer at 5000 rpm, resulting in a resist thickness of 3 µm. The temperatures for pre‐ and post‐baking of the photo resist are 90 and 110 °C for 1.5 min, respectively. Using the pre‐fabricated alignment marks and mask aligner for photolithography (EVG620, EVG), a pattern of the driving electrode was transferred to the coated photo resist. The pattern was developed in tetramethylammonium hydroxide (NMD‐W, Tokyo Ohka Kogyo Co., Ltd.) for 2.5 min. Then, similar to etching the silicon device layer, the silicon substrate layer was etched to form the driving electrode. The 2D structure on the device layer was released from the substrate layer by buffered hydrofluoric acid etching of the silicon dioxide layer. Finally, the substrate was dried by using a supercritical dryer.

All the topological couplers for this work were measured in a high‐vacuum chamber under 2.0 × 10^−5^ Pa to suppress the effect of viscosity damping in atmosphere. Contacting the driving electrode and device layer electrically by manual‐probes, the mechanical vibration was excited at the end of the V‐shaped waveguide by a sinusoidal signal with a function generator (RIGOL, DG972), stabilized power supply (Kikusui Electronics Corporation, PMX110‐0.6A), and bias tee (Tektronix Keithley Instruments, PSPL5530B). For the excitation of signals, the signal amplitude of *V*_pp_ = 5.0 V and the direct voltage of *V*_dc_ = 40 V were set, respectively. The vibration velocity along the in‐plane direction on each unit cell was measured by a laser doppler vibrometer (Ono Sokki, LV‐1800). A lock‐in amplifier (NF Corporation, LI5660) filters and amplifies output signals from the laser doppler vibrometer. Then, a digital oscilloscope (Tektronix, MSO64B) displays vibration velocity at the measurement point in the 2D structure. The measurement position was controlled by using controllable four‐axes piezo stages (Sigmakoki, VSGSP60(XY), VSGSP60(Z), and SHOT‐304GS).

### Bit Error Rate and Channel Capacity

The BER with the topological coupler was evaluated by output signals *E_p_*/*E*_1_ as shown in Figure 4c. As preparation of calculation of BER, the output signal was expressed from each port for digital signal *d*  =  0,  1 as *E*_*p*, *d*_(*n*)/*E*_1_(*n*). Average μ_*p*, *d*_ and variance $\sigma_{p,d}^{2}$ of *E*_*p*, *d*_(*n*)/*E*_1_(*n*) were calculated as $\mu_{p,d}=\left(1/N\right)\sum_{n=0}^{N}\left\{E_{p,d}\left(n\right)/E_{1}\left(n\right)\right\}$ (*N*  =  3.25  × 10^7^) and $\sigma_{p,d}^{2}=\left(1/N\right)\sum_{n=0}^{N}{\left[\left\{E_{p,d}\left(n\right)/E_{1}\left(n\right)\right\}-\mu_{p,d}\right]}^{2}$, respectively. Defining the variable parameter *X*_*p*, *d*_ along the axis of *E_p_*/*E*_1_ in Figure 4c, the probability density function was described *p*(*X*_*p*,*d*_) as $p\left(X_{p,d}\right)=\left(1/\sqrt{2\pi }\sigma_{p,d}\right)\exp\left\{-{\left(X_{p,d}-\mu_{p,d}\right)}^{2}/2\sigma_{p,d}^{2}\right\}$. As one example of BER calculation, when the bit error rate was evaluated for Δ*f*  =  45 kHz at port 2(3) without mitigating the effect of frequency transition, first the threshold ζ_2(3)_(Δ*f*) = 0.795(0.189) was calculated minimizing the error in the overlapping area of *p*(*X*_2(3),0_) and *p*(*X*_2(3),1_), i.e., $\zeta_{p}=\mathrm{argma}x_{\zeta_{p}}\left[P\left[{\hat{d}}_{p}=1|d_{p}=1\right]+P\left[{\hat{d}}_{p}=0|d_{p}=0\right]\right]$. Here, $P[{\hat{d}}_{p}=1|d_{p}=1]$ represents the probability that the obtained signal was “1” when the incoming signal was “1”. Similarly, $P[{\hat{d}}_{p}=0|d_{p}=0]$ represents the probability for correct reception and demodulation of digital signal “0”. The expression of $\mathrm{argma}x_{\zeta_{p}}\left[F\right]$ denotes the value of ζ_*p*_ for the maximum *F*. With threshold ζ_*p*_ and *p*(*X*_*p*,*d*_), bit error rate at each port *BER_p_* was given by

(1) $$\begin{array}{ccc} BER_{2} & = & \frac{1}{2\sqrt{2\pi }\sigma_{2,0}}\int_{0}^{\zeta_{2}}\exp\left\{-\frac{{\left(p\left(X_{2,0}\right)-\mu_{2,0}\right)}^{2}}{2\sigma_{2,0}^{2}}\right\}dX_{2,0}\\ & & +\;\frac{1}{2\sqrt{2\pi }\sigma_{2,1}}\int_{\zeta_{2}}^{\infty }\exp\left\{-\frac{{\left(p\left(X_{2,1}\right)-\mu_{2,1}\right)}^{2}}{2\sigma_{2,1}^{2}}\right\}dX_{2,1} \end{array}$$

(2) $$\begin{array}{ccc} BER_{3} & = & \frac{1}{2\sqrt{2\pi }\sigma_{3,1}}\int_{0}^{\zeta_{3}}\exp\left\{-\frac{{\left(p\left(X_{3,1}\right)-\mu_{3,1}\right)}^{2}}{2\sigma_{3,1}^{2}}\right\}dX_{3,1}\\ & & +\;\frac{1}{2\sqrt{2\pi }\sigma_{3,0}}\int_{\zeta_{3}}^{\infty }\exp\left\{-\frac{{\left(p\left(X_{3,0}\right)-\mu_{3,0}\right)}^{2}}{2\sigma_{3,0}^{2}}\right\}dX_{3,0} \end{array}$$

The theoretical limit of transmission speed in the topological coupler was evaluated by maximizing the mutual information, i.e., channel capacity.^[^ ^64^, ^68^, ^69^ ^]^ Mutual information was expressed as $I\left(d;\;\hat{d}\right)=H\left(\hat{d}\right)-H\left(\hat{d}|d\right)$, where *H*(*Y*) is the entropy of random variable *Y*. For binary communication such as the demonstration, the entropy is *H*(*P*) = −*P*_0(1)_log _2_ *P*_0(1)_ − (1 − *P*_0(1)_)log _2_ *P*_0(1)_, where two error possibilities have the forms of $P_{0}=P\left[\hat{d}=1|d=0\right]$ and $P_{1}=P\left[\hat{d}=0|d=1\right]$, respectively. $I(d;\;\hat{d})$ could be maximized when d*I*/dα = 0, where α = {1 − *P*_1_(1 + *z*)}/{(1 − *P*_0_ − *P*_1_)(1 + *z*)} and $z=2^{\left\{H\left(P_{0}\right)-H\left(P_{1}\right)\right\}/\left(1-P_{0}-P_{1}\right)}$. Finally, using α, the channel capacity *Ch* was calculated as follows:

(3) $$Ch=\log_{2}\left(1+z\right)-\frac{1-P_{1}}{1-P_{0}-P_{1}}H\left(P_{0}\right)+\frac{P_{0}}{1-P_{0}-P_{1}}H\left(P_{1}\right)$$

Note that the BER and channel capacity for the insets in Figure 4d,e are calculated by using μ_*p*, *d*_ and σ_*p*, *d*_ after elimination of the signal transition from 0 (1) to 1 (0).

## Conflict of Interest

The authors declare no conflict of interest.

## Author Contributions

K.F. performed simulations, fabrications, experiments, and analysis. K.Y. supported the reinforcement of theoretical aspects, and analysis of simulations. H.T. supported the analysis of experimental digital signal transfer for estimating the communication performance. H.I. contributed to discussions on theoretical and numerical analysis and supervised this project. All authors contributed to discussions and manuscript preparation.

## Supporting information



Supporting Information

## Data Availability

The data that support the findings of this study are available from the corresponding author upon reasonable request.
