# Supplementary material for: Quantum Valley Hall Effect‐Based Coupler with Continuously Tunable Transmission for Topological Information Communication
Source: Adv Sci (Weinh). 2025 Jun 29;12(35):e06732. doi: 10.1002/advs.202506732 (PMC12462939; doi:10.1002/advs.202506732)
Supplement: Supplementary file 1 — Supporting Information [file ADVS-12-e06732-s001.pdf]

ADVANCED SCIENCE
Open Access

# Supporting Information

for *Adv. Sci.*, DOI 10.1002/advs.202506732

Quantum Valley Hall Effect-Based Coupler with Continuously Tunable Transmission for Topological Information Communication

*Keita Funayama*, Kenichi Yatsugi, Hiroya Tanaka and Hideo Iizuka*

Supporting Information

**Quantum valley Hall effect-based coupler with continuously tunable transmission for topological information communication**

*Keita Funayama*, Kenichi Yatsugi, Hiroya Tanaka, Hideo Iizuka*

**Output signals at the isolated port (port 4) for the bridge and zigzag boundaries-based couplers**

**Figure S1(a)** and **S1(b)** show the kinetic energy at port 4 for the topological couplers based on the bridge and zigzag boundaries shown in Figure 1 in the main manuscript, respectively. The energy in Figure S1 is normalized by the input energy at port 1, i.e. $E_4/E_1$, similar to the energy at other output ports as shown in Figure 1. In both couplers, the propagating kinetic energy from port 1 to port 4 is negligibly small for the low (red circles) and high (blue crosses) frequencies. The energy isolation at port 4 indicates that the waves propagating along the waveguides in the couplers are topologically protected.

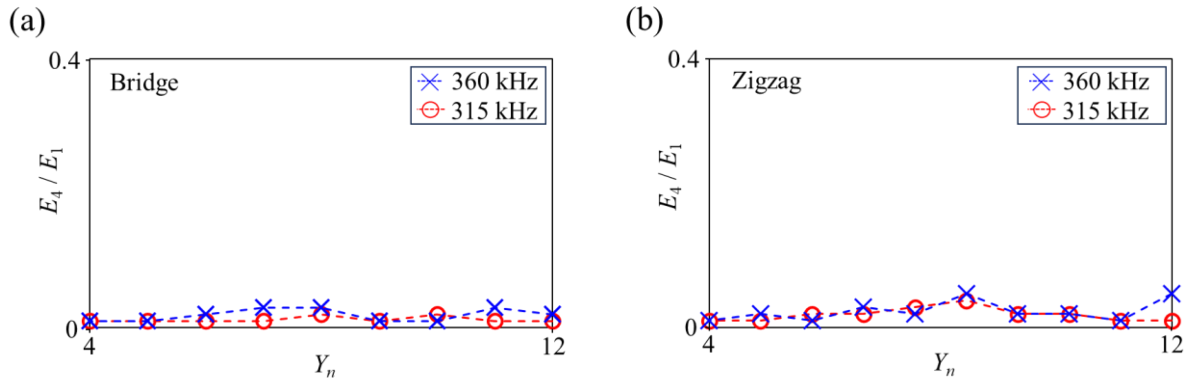


**Figure S1.** Output kinetic energy at port 4 for the topological couplers. a, b) The normalized kinetic energy at port 4 ($E_4/E_1$) for the couplers based on the bridge (a) and zigzag (b) boundaries. The circular and cross symbols represent the energy profiles for low and high frequencies, respectively.

**FSK signals in the topological coupler based on the zigzag boundaries**

We investigate digital signal transfer in the topological coupler consisting of the zigzag boundaries. Similar to the demonstration of the bridge boundaries-based coupler, we input the digitally modulated signal by frequency shift keying at port 1 as shown in **Figure S2(a)**. **Figure S2(b)** shows the kinetic energy at ports 2 (magenta) and 3 (cyan) as a function of time, respectively. As discussed in the main manuscript, the zigzag boundaries-based topological coupler is independent of the frequency, resulting in constant coupling between the V-shaped and straight waveguides. In the demonstration of digital data transfer, we see that output signals are constant at ports 2 and 3 regardless of the shift of input frequency. Thus, the zigzag boundaries-based topological couplers do not distinguish the frequency-modulated signal, while the coupler has a merit of robustly constant signal transfer under fluctuation in the incoming signal frequency for other application scenarios.

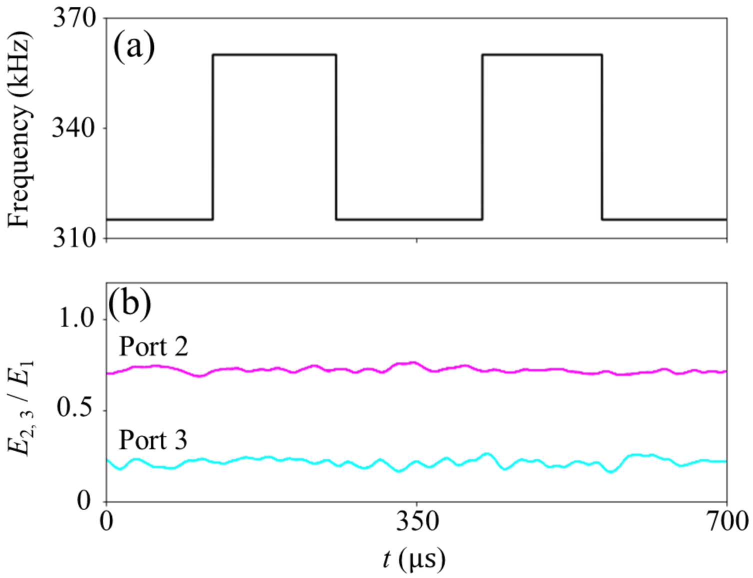


**Figure S2.** Digital data transfer in the zigzag boundaries-based topological coupler. a) Frequency of the input signal at port 1 as a function of time. b) Output kinetic energy at ports 2 and 3 as a function of time.
